# The Elementary Particles of Quantum Fields

**DOI:** 10.3390/e23111416

**Published:** 2021-10-28

**Authors:** Gregg Jaeger

**Affiliations:** Quantum Communication and Measurement Laboratory, Department of Electrical and Computer Engineering and Division of Natural Science and Mathematics, Boston University, Boston, MA 02215, USA; jaeger@bu.edu

**Keywords:** quantum field theory, particle, quantum field, interpretation, reductionism, ontology

## Abstract

The elementary particles of relativistic quantum field theory are not simple field quanta, as has long been assumed. Rather, they supplement quantum fields, on which they depend on but to which they are not reducible, as shown here with particles defined instead as a unified collection of properties that appear in both physical symmetry group representations and field propagators. This notion of particle provides consistency between the practice of particle physics and its basis in quantum field theory.

## 1. Introduction

The existence of elementary particles has come into question even though they are basic referents of particle physics, one of the most prominent fields of contemporary science, and though they are part of its most successful grand model of fundamental physics, the Standard Model. This is primarily because such particles have been considered simple quanta, that is, freely propagating excitations of quantum fields, which they are not when in the presence of interactions, which are omnipresent (cf., e.g., [1]). A commonly invoked analogy, based in straightforward mathematics, is that quanta are to quantum fields as harmonic vibrations are to the strings of a plucked stringed instrument, where only the strings exist and all vibrations are entirely reducible to their unforced motion. However, the elementary particles of particle physics are never strictly free but are only approximately free; particles, in general, are incessantly interacting, a situation in which they cannot be identified as simple excitations of a free field, even though such quanta have proven to be valuable tools of calculation. (For example, Brigitte Falkenburg has commented, “operationally, it is by no means possible to resolve [intermediary particles] into single particle contributions. They are nothing but the mathematical contributions to an approximation procedure: like the harmonics of the oscillations of a mechanical string, the Fourier components of a classical electromagnetic field, or the cycles and epicycles in Ptolemy’s planetary system“ ([2], p. 234)).

The question of the relationship between particle and field aspects of elementary systems in quantum field theory is thus both similar and different from the long-contemplated question of whether physical systems might behave as particles or waves as historically considered in basic quantum mechanics, where those have been either considered equally significant and complementary aspects or, alternatively, associated with competing alternate models of that theory: In quantum field theory, it has traditionally been most often considered that elementary particles are simply reducible to fields of quantum field theory rather than being either of equal ontological significance or non-existent. As is shown below, the relationship between particles and fields is more nuanced.

In a relativistic quantum field theory (RQFT) including interactions such those of the Standard Model (SM), the reduction of particles to fields as standardly suggested is impossible because the field-associated operators necessary to capture particulate nature are not well defined in general: Beyond their strong differences from classical particles, for example, regarding localization and number conservation (cf., e.g., [3]), quanta as formally indicated via the creation and annihilation operators and counted by field-mode occupancy are ill-defined because the very relativistic “number” operators needed to count them fail to exist for interacting fields, as discussed here in Section 3 (cf. [1,2], Ch. 6). The Standard Model of particle physics encompasses all recognized elementary particles, provides an account of the sorts and manners of interaction between them and succeeds predictively with unprecedented precision mainly through the use of the perturbative methods of scattering theory although, notably, the SM does not encompass the gravitational force. The most well confirmed component of SM is quantum electrodynamics (QED), which involves several sorts of particle, but a Klein-Gordon model of RQFT will be that primarily considered here for simplicity and clarity because it is a standard reference model for RQFT in relation to those of the SM, in turn, because it captures the elements of the SM that are salient to the problem at hand. (And, like QED, it is renormalizable, relieving us of that concern as well (cf., e.g., [4,5])).

The most common view of the ontology of RQFT recently has been that it includes only fields: Relativistic quantum fields are considered fundamental, and their amplitudes are always well defined in the interacting case; particle-quanta, if accepted at all into the ontology of RQFTs, are considered entirely reducible to fields and well defined only in those limited situations in which the Hilbert-space operators counting them are well defined, that is, in interaction-free limits (cf., e.g., Refs. 10–21 of [6]). For example, Steven Weinberg made the following claims.

“The Standard Model is a quantum field theory. The fundamental ingredients of nature that appear in the underlying equations are fields: the familiar electromagnetic field, and some twenty or so other fields. The so-called elementary particles, like photons and quarks and electrons, are ‘quanta’ of the fields—bundles of the fields’ energy and momentum. The properties of these fields and their interactions are largely dictated by principles of symmetry, including Einstein’s Special Principle of Relativity, together with a principle of ‘renormalizability,’ which dictates that the fields can interact with each other only in certain specially simple ways. The Standard Model has passed every test that can be imposed with existing experimental facilities” ([7], pp. 59–60); “By the mid-1970s it had become clear that the properties of these particles and all other known particles could be understood as mathematical consequences of …the Standard Model. The fundamental equations of the Standard Model deal not with particles and fields, but with fields of force alone; particles are just bundles of field energy” ([7], p. 109); “a(p) and $a^{†}\left(p\right)$ respectively annihilate and create a particle of momentum p [in free-field theories]. This is what we mean when [we] refer to elementary particles being bundles of the energy and momentum in some field”.

([8], p. 259)

Thus, the elementary particles have been viewed as derivative because quanta as standardly defined are entirely reducible to quantum fields, and talk of elementary particles has been considered superfluous among theorists, being for them merely a form of verbal short-hand, a *façon de parler* used mainly for convenience and used more frequently in discussions of experiments than in field theory.

The above view would appear to accord with the dictum that one should not postulate existents unnecessarily. But, particles, properly defined, have unique predictive value. Indeed, it has been understood since the discovery of the photoelectric effect that accounting for microphysical properties requires fields that differ fundamentally in character from classical fields; one of the physical novelties of quantum fields is their non-reductive support for at least some empirically confirmed *particle-like* behavior. This need is a primary reason that particles are still referred to in discussions and explanations in quantum mechanics and particle physics. And, the rejection of the standard quantum particle notion by many, which has taken place despite ongoing mention of particles in almost all experimental practice and much theoretical development, is due to their being something other than such simple field harmonics (or collections thereof) during interaction [1].

A consistent characterization of quantum particles that includes those characteristics differentiating them from both classical particles and simple field quanta is offered here, namely, that they are irreducible units of compresent, conserved properties that supplement relativistic quantum fields. The properties include the characteristics of Weinberg’s “bundles,” units of property sets associated with mass-energy. (This is not to be confused with Meinhard Kuhlmann’s far different, trope notion of quantum particle to which they term “bundle” has been applied [9]). I argue here that the rest mass, spin and charge, as the required elements of a unified, compresent set of properties provides the most proper definition of the elementary quantum particle associated with a given field; although elementary particles clearly do depend on fields, quantum particles are not quanta as standardly defined nor fully reducible to them when interacting but rather property bundles that are not defined via the annihilation and creation operators a(p) and $a^{†}\left(p\right)$.

Thus, an explication of the elementary particle notion is provided here that captures its quantum characteristics but does not suffer from the shortcomings of its predecessors that have led to skepticism regarding the existence of elementary particles. Such particles are shown to be non-trivial elements of the ontology of RQFT in Minkowski space-time. In addition to fitting current practice and theory—despite the common but inappropriate identification with the simple field excitation—this particle notion also accords with the long-held view of elementary forces as involving the exchange of properties of particles; it is in this realm where the non-reducibility of particles to fields becomes evident and why the notion of virtual particles as traditional particles has often been considered suspect [10,11]. For example, Heisenberg wrote “There is no description of what happens to the system between the initial observation and the next measurement.…The demand to “describe what happens” in the quantum-theoretical process between two successive observations is a contradiction in adjecto, since the word “describe” [or “represent”] refers to the use of classical concepts, while these concepts cannot be applied in the space between the observations; they can only be applied at the points of observation” (cf. [12], Section 2). The notion of particle as the simple quantum field excitation appearing elegantly in the Fock representation, is reviewed in Section 2; the critique of this notion is briefly reviewed in Section 3. The novel particle notion introduced in Section 4 as a precisely specifiable set of compresent properties according with the behavior of the quantum fields without being reducible to them, that is, as supervening on them as explicated in Section 5.

## 2. Elementary Particles and Their Fields

The defining properties of the elementary particles of RQFT are here indicated with the aid of the standard method of formal classification, Wigner’s group-theoretical approach in terms of the irreducible representations of the physical space-time symmetry group [13]. These symmetries have been required of the fields of RQFT appearing in the Lagrangian density of the Standard Model that are requirements imposed by relativity. The particles are defined here directly via the mass and spin values associated with distinct continuous unitary irreducible representations: The independent irreducible such representations are distinguished by different, fixed values of mass and spin (or parity), which correspond to the two Casimir invariants, $C_{1},C_{2}$; these values correspond to those empirically verified in experiments involving fundamental interactions.

In Minkowski space-time, to which attention is restricted here, physical particle classes correspond to the irreducible projective representations of the (restricted) Poincaré group of space-time symmetries, ISO${}^{+}\left(1,3\right)$ (cf., e.g., [14], Ch. 2, [15], Section 2.7), for transformations $x\to x^{\prime }=\Lambda x+a$. Such a group representation is given, for example, by the unitary continuous U(a,Λ) action of the (restricted) Poincaré group on a Hilbert space H such that

(1) U(a,Λ)U(b,M)=ωU(Λb+a,ΛM),

where $a,b\in R^{4}$, Λ and *M* are Lorentz matrices, and ω is a scalar. (The irreducibility of representations precludes the reduction of particles to constituent particles similarly defined, in accordance with their elementary character.) In the case of systems described by free-Lagrangian densities, those for which the corresponding field equations are linear, each representations typically takes the form of a single-particle Hilbert space from which a Fock space of multiple particles is constructed, and it is required that there be a unitary operator U(a,Λ) for each such space-time transformation such that $U\left(a,\Lambda \right)\phi \left(x\right)U^{†}\left(a,\Lambda \right)=\phi \left(\Lambda x+a\right)$, in order to guarantee the existence of desired canonical transformations between between coordinates of creation and annihilation operators for quanta of a field ϕ(x) quantized on the $t^{\prime }$-constant hyperplane (cf., e.g., [16], Section 3.1).

Nonetheless, elementary particles are *not* here taken here to be the standard field *quanta* defined in the Fock–Hilbert space constructed via the system free Hamiltonian but rather are indicated directly by the quantities that appear as Casimir invariants in such constructions because the general case of interest is not that of free systems but rather *interacting ones* in which such quanta are not particles. However, the interacting theory may still be illuminated by the formal structures developed for the corresponding free version theory, as seen in later sections. Specifically, particles are taken here to correspond directly to particle (rest) mass *m* and spin *s* (if $m^{2}$ is non-negative) which are also associated with the irreducible group representations discussed above, where the allowed spin values are integral or half-integral. (The integral and half-integral cases are those of the bosons and fermions, respectively. Spin corresponds to the spatial rotation group symmetry SU(2), although if m=0 the SU(2) symmetry does not relate to it). These invariant quantities represent, together with charges attributed by the SM, the physical properties of particles that propagate together as irreducible units in accordance with local causality in accordance with that of the fields on which they depend (see Equation (3) below); those of interest are those according with the properties of the particles of the SM, which are physically accepted due to its explanatory success. As discussed in detail in Section 4 below, such particles supervene on the quantum fields that appear in the Lagrangian density, which is invariant under these symmetry transformations, and their corresponding equations of motion are covariant under them. (However, the approach taken here is not one in which quantum particles are considered “emergent” and identified in “QFT Hilbert spaces in which the states have particulate properties” such as localization in the non-relativistic or a scattering limit where only states of free QFTs are considered; cf. [17], Section 2.6).

In the description of local field theories, one deals with quantities that are integrals over location $\vec{x}$ of functions that are point-wise defined, that is, functionals. Most important, the Lagrangian for the field has the form $L\left(t\right)=\int d^{3}\vec{x}L\left(\vec{x},t\right)$, the integrand being the Lagrangian density. The local field ϕ(x) operator (where $(\vec{x},t)=x$) of the Lagrangian formulation—i.e., the formulation by quantizing a given field Lagrangian density of a RQFT not including interactions—can be viewed as the Fourier transform of creation $a^{†}\left(\vec{p}\right)$ and annihilation operators $a(\vec{p})$ (for the creation of quanta) applicable to the system state given via just such a Fock Hilbert space construction, F, which naturally accounts for the symmetries of system states of various levels of excitation (‘occupation’) by quanta in the no-interaction case. And, for free fields, a Fock space of system states can be obtained by taking single-particle states as basis vectors for a Hilbert space in which quanta can be defined as excited states via the Fock space construction.

The standard (Hamiltonian) construction for a quantum-field takes a classical field together with a postulated canonical commutation relations for the local field ϕ(x) and its conjugate momentum $\pi \left(x\right)=\frac{\partial L}{\partial (\partial_{0}\phi \left(x\right))}$ (i.e., $\left[\phi \left(\vec{x},t\right),\pi \left(\vec{y},t\right)\right]=i\delta^{3}\left(\vec{x}-\vec{y}\right),\;\left[\phi \left(\vec{x},t\right),\phi \left(\vec{y},t\right)\right]=0$, $[\pi \left(\vec{x},t\right),\pi \left(\vec{y},t\right)]=0$) and identifies a lowest energy (‘vacuum’) state, i.e., considered to be without any excitation. When only free fields are considered, this state is |0〉 and the system state can be that of a number of quanta which propagate through space-time as plane waves or superpositions thereof (see Equations (5) and (6), below); the total number of quanta of the corresponding class present is represented using the product of such a corresponding operator, $N_{\mathrm{free}}$, connected with the total energy via the Hamiltonian and having eigenstates |n〉 corresponding to a counting number *n* of excitations. (And, in the free-field case, the total number of quanta is a conserved quantity; ibid.) Some elements of the mathematical structure are also useful in various analyses of processes when interactions are present, but this method cannot directly provide a particle correspondent for theories that include interactions. Indeed, this is one of the indications that the standard notion of particles directly identifiable with such freely propagating field excitations is problematic, as discussed in the following section (cf. [1]).

Although it is in the free field case that particles are *directly discernible* because it is only there that they are *isolated*, such isolation is rarely, if ever strictly present in the physical world due to the omnipresence of fundamental interactions. RQFTs that include interactions are typically defined via Lagrangian densities standardly written in terms of fields ϕ(x), their space-time derivatives, and couplings *g* of any interaction between them, which guarantees Lorentz invariance. The *causal* Lorentz-invariant theories of interacting particles in Minkowski space-time are just those local QFTs with Langrangians which are not purely quadratic in the field operators (cf., e.g., [18], Ch. 3). This is clear in the context of use of the S-matrix, *S*, of scattering scattering theory for the study of their behavior, the most successful predictive method of RQFT which is central to the practice of particle physics: *S*, a unitary matrix connecting initial, pre-scattering system states (defined in Hilbert space) with final, post-scattering states is Lorentz invariant if there is a set of operators satisfying the commutation relations of the Poincaré group, and these emerge naturally when the Lagrangian density itself is Lorentz-invariant [19].

Because the study of scattering for particles of the Standard Model is rather intricate—although it has an elegant Lagrangian and possesses the required symmetry properties—and because all of its aspects are not necessary to address the issues at hand here, the focus of our discussion will be primarily on the Klein-Gordon (KG) model, a simpler, representative one that captures the basic features involved in its full expression as a QFT (cf., e.g., [20], Ch. 20–22); the KG model serves as the best generic RQFT because every component of *any* quantum field is required to satisfy the free Klein-Gordon eq. cf., e.g., [15], p. 200). (However, it should be recognized that the QCD component presents challenges regarding renormalization that are not addressed here). The Lagrangian density for a KG model—here taken to be real rather than complex for further simplicity which leaves aside explicit antiparticles—supporting massive scalar particles is

(2) $$L\left(x\right)=-\frac{1}{2}{\left(\partial_{\mu }\phi \left(x\right)\right)}^{2}-\frac{1}{2}m^{2}\phi {\left(x\right)}^{2},\;$$

which serves here as our basic theoretical reference function. Writing the action as the space-time integral of the Lagrangian density and imposing the condition that the action be stationary yields the relativistically covariant equation of motion for the field according to the Action Principle (cf., e.g., [14], Ch. 3). In this case, one obtains equations of motion for the field explicitly satisfying relativistic energy-momentum conservation analogously to the expression for ordinary particles: $\left(\partial_{\mu }\partial^{\mu }+m^{2}\right)\phi \left(x\right)=0$. Although the freely propagating field disturbances of models not including interactions that correspond to quanta take the form of spatial plane waves or superpositions thereof, describable via corresponding propagators, more general field disturbances arise that are standardly and accurately described in perturbation-theoretical treatments via (more general) propagators when interactions are present, the case of general interest.

The interacting-field generalization of the Lagrangian density of a free-theory of our example includes an additional term, $L_{I}$, involving a coupling constant, λ, for interactions and the fields that interact; an example commonly considered is $L_{I}=-\frac{\lambda }{4!}\phi {\left(x\right)}^{4}$, often referred to simply as $\phi^{4}$-theory. (The Hilbert-space description can be derived from generalized Green’s functions for the theory when desired (ibid.)). The locality of interactions is enforced directly at the field level of description by the requirement that the interaction term $L_{I}$ be local,

(3) $$[L_{I}\left(x\right),L_{I}\left(y\right)]=0\;,$$

if ${\left(x-y\right)}^{2}<0$; this itself is so by virtue of the condition that the field commute with itself at space-like separations, that is, the micro-causality condition that [ϕ(x),ϕ(y)]=O if ${\left(\vec{x}-\vec{y}\right)}^{2}<0$. More generally, the latter condition precludes signaling between points of space-time, *x* and *y* because it enforces the commutativity of the field operators associated with any pair of space-time locations x,y for any well-defined measurable field or particle quantity at space-like separated distant locations (cf., e.g., [21], Ch. 9). Operationally, this condition requires that the time-evolved versions of the operators for physical quantity commute with the projectors onto the eigenstates for their allowed values when measurements are made at distinct space-like separated locations (cf., e.g., [22]).

The probabilities of the various interaction processes constituting a great deal of the empirical content of a QFT are obtainable by integration over a range of parameters of the propagators along with other factors to provide the corresponding probability amplitudes for scattering state transitions and so expectation values of scattering matrix *S*. Such calculations are standardly carried out through the use of the Feynman rules for the interaction terms appearing in theory’s Lagrangian, which involve the propagators and field-particle coupling constants of associated diagrams. Before examining the relation of those probabilities to elementary particle processes, let us first consider briefly the failure of the standard alternative, the identification of particles with elementary field excitations, i.e., quanta.

## 3. Particles Are Not Simple Field Quanta

While the remarkable power of the Standard model for predicting the outcomes of particle-physics experiments conceived of as involving the scattering of subatomic particles is evident, the traditional approach to conceiving elementary particles, as field quanta, has fared badly under close scrutiny. For example, Doreen Fraser has provided a clear, broad-context critique [1] of the thesis that such quanta are fundamental to the ontology of QFT, in particular, in one its most valiant defenses, given by Jonathan Bain [23], that attempts to provide finite-time *states* of an interacting system in terms of those of infinite-time limits; this critique pointed out that the domain of application of the concept is too narrow for such particles to be admitted in interacting RQFT ([1], p. 842).

“The point at issue is whether entities with certain properties—particle-like properties—exist. More precisely, does QFT support the inclusion of particle-like entities as fundamental entities in our ontology? Bain contends that the fact that there is no free field total number operator $N_{\mathrm{free}}$ at intervening times is irrelevant. But, since the question is whether there are entities with particle-like properties at finite times, what is certainly relevant is whether the interacting system contains entities that are countable. In terms of the formalism, it is relevant whether a surrogate for $N_{\mathrm{free}}$ is available at finite times. … The ‘quanta’ in Bain’s scheme may not possess any particle-like properties in the presence of interactions. Appealing to the theory of a free system with which the interacting system is associated in an idealized infinite past or future does not fill this gap in the evidence”.

([1], pp. 856–857)

The focus of this critique is the unavailability of an operator $N_{\mathrm{free}}$ for the total number of quanta at finite times in interacting RQFT for which eigenstates could be considered [1]. Fraser notes that the crux of the problem is “the quanta interpretation that naturally arises from the Fock representation for a free system,” and her arguments “establish that it is not possible to extend this quanta interpretation to an interacting system” ([1], pp. 856–857). In particular, a “Hilbert space representation cannot be constructed by Fourier decomposing a classical interacting field because the resulting expressions are not relativistically covariant, and therefore are not candidates for physical fields in relativistic QFT” (ibid.).

An additional difficulty, unrelated to relativistic considerations, arises for the construction of well-defined number operators in the interacting field case, namely, the lack of availability of the Fourier transform needed to provide the required creation and annihilation operators from which a number operator is constructed, as $N\left(\vec{p}\right)=a_{\vec{p}}^{†}a_{\vec{p}}^{}$ (either by Fock-space method discussed in the following section or by another method analogous to it). In the free case, the states of definite numbers of quanta can be written as successive applications of $a_{\vec{p}}^{†}$ to the vacuum state |0〉; there, crucially, the energies of the number states correspond, as they must, to solutions of the classical field equation via an appropriate Fourier decomposition in terms of solutions of the Klein-Gordon equation having the form $e^{ik\cdot x}$ with k=m. However, the solutions for the corresponding interacting field equation are not of this required form. (For further details regarding the difficulties of construction such number operators, see Section 4 of [1] and Section 4).

Regarding attempts to circumvent these difficulties by finding a mathematical analogue to Fock-space formulation, Fraser commented that

“Instead of Fourier decomposing the classical interacting field into functions of the form $e^{ik\cdot x}$, one might attempt to decompose it into functions of some other form. A suggestion along these lines is mooted in Huggett …. [who] floats—but ultimately rejects—the possibility of extending the oscillator analogy to the interacting case in the following way: ‘[f]or an interacting field the oscillators do not move independently, but as if they were interconnected: there might be further springs, one between any pair of bobs’ (p. 628). Translated into the terms of the present discussion, the suggestion is that instead of decomposing the field into independent oscillators—the plane waves $e^{ik\cdot x}$—the field should be decomposed into coupled oscillators, which are represented by functions of some other form”.

([1], p. 852)

Fraser then points out two difficulties with this suggestion which “are both substantial“: (i) finding such functions, and (ii) satisfying Lorentz covariance, and concludes that “it seems safe to conclude that it is not possible to obtain an analogue of the Fock representation suited to an interacting field by applying an analogue of the mathematical construction that produces the Fock representation for a free field” (ibid.).

Along with this critique, Fraser indicated two responses to the situation encountered by the standard explication the particles of QFT just discussed:

One route is to “agree that quanta are not part of the ontology of fundamental entities described by QFT, but to argue that nevertheless quanta do exist according to QFT. For example, Wallace considers the ‘particle’ concept to be an emergent concept. For this to be a viable response, the cogency of the distinction between fundamental and less fundamental entities must be defended and a case must be made for admitting additional, non-fundamental entities into our ontology. Alternatively, the quanta concept may be regarded as a concept that is only approximately or ideally applicable because it is restricted to free systems. In the context of a scattering experiment, free systems occur in the idealized limits of infinitely early and late times.”

([1], pp. 857–858)

As shown below, the particle notion advocated here is not that of the “quanta concept” and does not suffer from the shortcomings of the standard quanta interpretation; it is more a response of the first suggested kind; its particles (though not such quanta) *are* ontologically necessary in the sense that they are not (entirely) reducible to fields and have an explanatory function not satisfied by fields.

The argument for their admission is exactly that they capture the quantum nature of interactions—the concomitant and discrete joint appearance of properties that propagate causally that are observed together in discrete units when measured for—something which not captured by the fields alone. By considering the standard method for predicting the results of observations of the elementary particles, scattering theory, it is shown below that RQFT involves particles that are well-defined objects supervening on the relativistic quantum fields and not simple field quanta (which would require well-defined number operators to exist for all times). Although they are not countable during intermediate times of interaction processes because quantum indeterminism precludes it, the defining properties of particles are determinate at all times, and other traditional particle properties are countable at the end of the overall interaction process.

The approach explicated here differs precisely in not offering an analogue of the Fock-space representation (or wave-like propagation); instead, it looks to the existing formal structure of (the scattering theory treatment of) RQFT to identify a relevant field structure that is also *not* required to reduce the particle properties; it is the field propagator, which is not function but a more general structure, a distribution to which harmonic functions may only incidentally correspond when interactions are not present. (In a later article after the appearance of Fraser’s critique, Bain similarly cautions against use of the standard quanta approach to quantum particles [24].) During interactions, the field propagators contain “off-shell” masses, often prompting the use of the term “virtual” for the particles involved; this term is eschewed here because all particles are then, strictly speaking, virtual except those which cannot possibly be interacting, a condition which is rarely, if ever, strictly satisfied; the connotation of the term is, therefore, false.

## 4. Propagators and Properties

The particle–field relationship, which is the dependence of particles on fields but with the former irreducible to the latter, consists fundamentally in common properties of mass and spin (or helicity). The commonality of the property of mass to both is evidence of the relation of particles and fields; but the value differences preclude an ontological reduction of particles to fields, and particles cannot be removed from the fundamental ontology of QFT as they could if they were identical to an element of the field, such as an excitations is. Let us now examine this relationship more carefully via the relevant formal elements—the first and foremost of these being the (two-point) correlation function, which allows for the explication of the relationship between particle-quantities and field-quantities in general via elements of the formalism applicable in the free case.

In the simple case of a system described by the interaction-free Langrangian density of Equation (2), this function is a propagator, the Wightman function

(4) D(x−y)=〈0|ϕ(x)ϕ(y)|0〉,

where |0〉 represents the lowest energy state of the system and *D* is a function of the distance x−y, guaranteeing space-time translation invariance; D(x−y) is a Green’s function in that it satisfies the inhomogeneous Klein–Gordon equation with the inhomogeneity δ(x−y). D(x−y) describes the free propagation of a particle of fixed mass *m* away from a point. In this free case, a connection can be made with the traditional notion of particles as field quanta because it is possible to identify the standard field quanta as particles propagating with definite momentum and having creation $a^{†}\left(\vec{p}\right)$ and annihilation operators $a(\vec{p})$ for quanta in a Fock space F, which naturally incorporates the properties of multi-particle states; it allows the fields to be written as momentum integrals over creation and annihilation operators for corresponding field excitations (i.e., quanta),

(5) $$\phi \left(\vec{x}\right)=\int \frac{d^{3}p}{{\left(2\pi \right)}^{3}}\frac{1}{\sqrt{2\omega_{p}}}\left(a_{\vec{p}}\;e^{-ip\cdot x}+a_{\vec{p}}^{†}\;e^{ip\cdot x}\right)\;,$$

where $\omega_{p}=p_{0}=E_{p}$ is the energy component of the four-momentum, *p*. There is also a well defined particle number operator $N_{\mathrm{free}}\left(\vec{p}\right)=a_{\vec{p}}^{†}a_{\vec{p}}^{}$ and the Hamiltonian $H_{0}=\int \frac{d^{3}p}{{\left(2\pi \right)}^{3}2\omega_{p}}a_{\vec{p}}^{†}a_{\vec{p}}^{}\omega_{p}$$=\int \frac{d^{3}p}{{\left(2\pi \right)}^{3}2\omega_{p}}N_{\mathrm{free}}\left(\vec{p}\right)E_{p}$, with $a_{\vec{p}}^{}|0\rangle =0$.

In particular, the function appearing in Equation (4) characterizes propagation of a quantum field contribution between the space-time points *x* and *y*, i.e., it is the simplest sort of propagator and describes propagation as a plane wave, i.e., via the form $e^{ik\cdot x}$:

(6) $$D\left(x-y\right)=\int \frac{d^{3}p}{{\left(2\pi \right)}^{3}}\frac{1}{2\omega_{p}}\exp^{-ip\cdot (x-y)}\;.$$

(The other pair of factors in the integrand corresponds to an invariant element of phase space; cf., e.g., [14], Section 4.1). If a timelike space-time interval of duration $t=x^{0}-y^{0}$ is considered, then one may obtain $D\left(x-y\right)∼e^{-imt}$ (cf., e.g., [5], pp. 27–28). When considered in the complex plane, this propagator possesses a pole at square of the particle rest mass $m_{\mathrm{rest}}$ identical to that of the KG particle appearing in the particle classification according to the group-theoretic method discussed in Section 2; a free such stable particle obeys the relation energy-momentum conservation requirement of Equation (2), and $k^{2}=m^{2}$ where *m* is equal to the real rest mass, $m_{\mathrm{rest}}$ (i.e., the propagator mass value is ‘on mass-shell’: $k^{2}=\omega_{p}^{2}-{\vec{p}}^{2}=m_{\mathrm{rest}}^{2}$). Unlike the case of particles of theories including interaction, free particles may be reduced to fields on-shell and, in principle, disregarded in purely theoretical physical descriptions; being so reducible, these particles might be considered redundant on the ontology of a model QFT in which no interactions take place between particles.

However, as discussed in broader context in the previous section, such a description and any ontological reduction of particle to field fail for QFTs when interactions are included; and so the traditional quanta ontology of RQFTs will fail as a fundamental ontology. In order to better understand particles in RQFT, it is helpful to look at successful physical practice involving them. Generally, the best empirical predictions for particle scattering resulting from particle interaction are found via perturbation theory, where interactions are considered to perturb *previously free propagation* of the large earlier-time limit, and a free propagation of resulting particles is assumed to take place again in the later large-time limit. As noted by James Fraser, “Philosophers have tended to shy away from perturbation theory in their investigations of QFT”. However, as he has also pointed out, “the perturbative framework should be understood as a method for producing approximations without addressing the project of constructing interacting QFT models”, for then “the mathematical sloppiness we do find in perturbative calculations in the physics literature becomes a less pressing foundational concern” and “Haag’s theorem is not as disastrous for the perturbative framework as it initially seems. In brief, the result does not undermine standard perturbative calculations because they do not posit the existence of a model satisfying the relevant set of inconsistent assumptions” [25]. The idea is to consider weaker interactions, i.e., those for which the interaction strength *g* (for our KG model, this is λ) is sufficiently small, to understand what takes place during interactions.

Here, our interest is in the relation of particle and field. We find that the propagator is the general correspondent in field-theoretical formalism of the particle—the field itself has a Hilbert-space operator as its correspondent—in that the propagator describes causal propagation of energy-momentum with an associated mass and spin (or helicity): The combination of properties appearing in it, common to both field and particle, allows for the formal relationship between the particle and the propagator (properly integrated over space and time), although there is, in general, a subtle relationship between the values taken. That this is possible for the case of interacting theories as well as the free case above is most evident in the description of scattering processes when studied via perturbation theory. There, the propagators naturally appear in the calculations for predicting and explaining scattering events, wherein these properties are conserved and particles are, in general, accordingly transformed into one another as seen by comparison of initial and final system states: When interactions are included, instead of the right-hand-side of Equation (4), the basic correlation function of interest is 〈Ω|T{ϕ(x)ϕ(y)}|Ω〉, where |Ω〉 is the lowest-energy state of the vacuum in this case which can differ from that of a strictly zero-occupancy state |0〉 as defined above. Field propagation in an interacting theory is also no longer always governed by a simple propagator of the form of D(x−y), and the solutions of the equations of motion will longer always correspond to plane-waves propagating as in the free-field case just described. One finds that, unlike approximately non-interacting particles—e.g., scattered, i.e., prepared and detected particles at large times—, the masses for particles mediating the interaction of two or more field-particles and for “dressed” particles appearing in the field expressions are off mass-shell: the corresponding field propagators have masses not corresponding to the particle mass indicated by the Wigner classification.

During field interactions, e.g., in a particle scattering situation, propagators have a form at best only *approximating* the form of Equation (6). In particular, the mass value appearing in the propagator is no longer fixed. The values it takes will also, in general, correspond to differently located poles when higher-order interactions take place. The set of possible interactions involves a non-trivial propagation topology which is captured by the time-ordered, or *Feynman (KG) propagator*, $\Delta_{F}\left(x-y\right)$, which accords with the requirement of relativity by appropriately combining Wightman functions. The perturbation-theoretical treatment of interaction allows $\Delta_{F}\left(x-y\right)$ to be used when defined in terms of the (free theory’s) lowest-energy state |0〉 even though it differs from the physical vacuum state |Ω〉:

(7) $$\Delta_{F}\left(x-y\right)=\langle 0|T\left\{\phi \left(x\right)\phi \left(y\right)\right\}|0\rangle \;,$$

where *T* is the time-ordering operator needed to satisfy the demands of relativity (cf., e.g., [5], Section 4.2). It is a solution of the inhomogeneous relation

(8) $$\left(p^{2}-m^{2}+i\epsilon \right)\Delta_{F}\left(x-y\right)=\delta^{\left(4\right)}\left(x-y\right)\;,$$

where ${\vec{p}}^{2}\neq \omega_{p}^{2}-m^{2}$ in general, implying a variable (‘off-shell’) mass $m\neq m_{\mathrm{rest}}$, which does not involve a simple wave form integrand, $e^{ip\cdot (x-y)}$ as in Equation (6), due to the inhomogeneity needed to include terms accounting for sources of influence on the field, precluding the analogue of Equation (5) for representation of the field operator; in the space-time representation, the two-point correlation function between two space-time points is of the form

(9) $$\Delta_{F}\left(x-y\right)=\int \frac{d^{4}p}{{\left(2\pi \right)}^{4}}\frac{e^{-ip\cdot (x-y)}}{p^{2}-m^{2}+i\epsilon }\;,$$

where ϵ>0 with the effect of displacing the poles, which would appear due to the form of the denominator were it allowed to take the value 0, from the real $p^{0}$ axis, and $\Delta_{F}\left(x-y\right)$ is evaluated in the limit ϵ→0 after integration has been performed; a simpler denominator with ϵ=0, namely $p^{2}-m^{2}={\left(p^{0}\right)}^{2}-{\vec{p}}^{2}-m^{2}$ would give rise to poles at $p^{0}=\pm \omega_{\vec{p}}=\pm \sqrt{{\vec{p}}^{2}-m^{2}}$ and an ill-defined integral.

During interactions that can be described as scattering processes, a factor contributing to the probability amplitude for an interaction-mediating disturbance from *x* at one time ($x^{0}$) and *y* at another time ($y^{0}$) is captured via $\Delta_{F}\left(x-y\right)$, which is a factor along with others enforcing momentum conservation in the amplitude density, which in turn is integrated over space-time to provide the corresponding amplitude; when squared this amplitude is the contribution to the scattering probability where the propagators appear. [Note that this treatment explicitly incorporates Lorentz-covariance, and $\Delta_{F}\left(x-y\right)=\Delta_{F}\left(y-x\right)$.] In situations of interaction, instead of a simple wave, a *disorderly field disturbance* appears in the integral required for the calculation of the possible transitions between initial and final system states, which will in general differ, and energy-momentum will be exchanged between particles of the initial state. [Note that this propagator is also nearly identical to that for a photon, except that in the free case it carries mass; cf. [5], Sections 4.8 and 9.4]. Cauchy’s residue theorem provides the tools for evaluating Equation (9), where two poles are present in the complex plane, one ϵ above the axis of negative real components and one ϵ below the axis of positive real components, in particular, at $p_{0}=\pm \left(\omega_{p}-i\epsilon \right)$: The integral of Equation (9) is evaluated by closing the corresponding contour about the pole of interest—that for the propagation underlying that of the particle, or that for the propagation underlying that of its anti-particle. [Note that the antiparticle may be identical to the particle and so not called so, cf., e.g., [18], Section 3.2]. When $x^{0}>y^{0}$ the integration over $p^{0}$ can performed by closing the contour of integration below the corresponding pole, and when $x^{0}<y^{0}$ above the corresponding pole.

It is important to note that, as mentioned above, the variable mass value in the propagator $\Delta_{F}$, appearing in its denominator is no longer the fixed rest mass, $m_{\mathrm{rest}}$. As can be seen by factoring the denominator of the integral of Equation (9), there are poles in the complex mass-squared (energy) plane both below the positive real axis and above it by the distance ϵ corresponding to its reaching zero; the origin of the difference, in general, between the mass of the particle responsible for the perturbation of the interacting free particles and the mass of the field propagator can be understood in perturbation-theory by considering the time-dependent interaction Hamiltonian, $H_{\mathrm{int}}$, corresponding to the interaction accounted for in the Lagrangian density in conjunction with the Fock space of the free theory. The treatment of the problem of the electromagnetic interaction between two massive particles due to the exchange of a zero-mass particle, the photon, by Aitchison and Hey ([26], Ch. 5), serves as a good example: It parallels the treatment of KG theory, which is our main reference model here (cf. the KG treatment of [27], Section 2.4), and provides a connection with the more general considerations taken up in the following section.

Consider, in particular, the second-order transition amplitude between incoming and outgoing states in an overall scattering process ($a+b\to a^{\prime }+b^{\prime }$) not for KG theory, but for the case of an *electromagnetic interaction between two scalar particles*. This is a vivid example because it involves a force mediating particle that is *massless* but where the field propagator involves a *non-zero* mass. The photon (having the standard mass m=0 attributed as per the Wigner method) can be viewed as the mediator of this interaction. The corresponding disturbance (i.e., of the electromagnetic field; cf. [26], Ch. 5.6) with the two possible time-orderings treated separately (so that each is non-covariant but when added will be covariant as in the Feynman propagator treatment of the KG model just considered above) and integration is performed over space:

(10) $$\begin{array}{ccc} A_{\mathrm{fi}}^{\left(2\right)} & = & -i\sum_{n}\left\{\int d^{3}x\langle p_{a}^{\prime }p_{b}^{\prime }|H_{\mathrm{int}}\left(\vec{x},0\right)|n\rangle \right\}\left(\frac{1}{E_{a}+E_{b}-E_{n}}\right)\\ & & \;\;\;\;\;\;\times \left\{\int d^{3}x^{\prime }\langle n|H_{\mathrm{int}}\left({\vec{x}}^{\prime },0\right)|p_{a}p_{b}\rangle \right\}2\pi \delta \left(E_{f}-E_{i}\right)\;, \end{array}$$

where |n〉 is the *n*-th excited free state of the system.

The delta functions here impose energy conservation, i.e., that $E_{a}+E_{b}=E_{a}^{\prime }+E_{b}^{\prime }$, while that quantity differs from $E_{n}$ (as it must for the middle factor of the integrand to remain finite) and $\left|n\rangle =\right|p_{a}^{\prime }p_{b}^{\prime };k,\lambda \rangle$ so that the sum over *n* is, in effect, one of all possible λ (helicity) and *k* (4-momentum) values (cf. [26], Eq. 5.160), and $E_{n}=E_{a}^{\prime }+E_{b}+E_{k}$. One then has

(11) $$\begin{array}{ccc} A_{\mathrm{fi}}^{\left(2\right)} & = & -i{\left(2\pi \right)}^{4}\delta^{4}\left(p_{a}^{\prime }+p_{b}^{\prime }-p_{a}-p_{b}\right)N_{a}N_{b}N_{a}^{\prime }N_{b}^{\prime }e_{a}e_{b}\\ & & \;\;\;\;\;\;\;\times {\left(p_{b}+p_{b}^{\prime }\right)}^{\mu }{\left(p_{a}+p_{a}^{\prime }\right)}^{\nu }\sum_{\lambda }\frac{\varepsilon_{\nu }^{*}\left(k,\lambda \right)\varepsilon_{\mu }\left(k,\lambda \right)N_{k}^{2}}{2E_{k}\left(E_{a}+E_{a}^{\prime }-E_{k}\right)}. \end{array}$$

The probability amplitude for the also possible, opposite time-ordering is just the same but with $E_{n}=E_{b}^{\prime }+E_{a}^{\prime }+E_{k}$ and polarization vectors ε and $\varepsilon^{*}$ are interchanged here. The sum of these two amplitudes complex-squared provides a probability for the scattering process to take place in via the photon. Due to overall energy conservation for the combination of these two amplitudes for the presence of a single-disturbance between the scattering particles, one finds then the crucial factor of the corresponding propagator

(12) $$\frac{1}{2E_{k}}\left(\frac{1}{E_{a}+E_{a}^{\prime }-E_{k}}-\frac{1}{E_{a}+E_{a}^{\prime }+E_{k}}\right)=\frac{1}{{\left(E_{a}-E_{a}^{\prime }\right)}^{2}-E_{k}^{2}},$$

from which, given the requirements of the delta functions in Equation (10), one has $E_{k}^{2}={\vec{k}}^{2}={\left({\vec{p}}_{a}-{\vec{p}}_{a}^{\prime }\right)}^{2}$. The quantity in Equation (12) is therefore

(13) $$q^{2}={\left(p_{a}-p_{a}^{\prime }\right)}^{2},$$

which is the *non-zero* mass appearing in the denominator of the electromagnetic field Feynman (covariant) propagator (the square of the four-momentum exchanged by the photon). It is, in general, different from that of the particle, m=0, assigned via the Wigner classification (cf. Section 2). The reduction of the photon to a field is thereby precluded.

## 5. Particles Supervene on Fields

The relation between particles (defined as a unified collection of properties according with space-time symmetry constraints) and fields is one of non-reductive dependence of the former on properties the latter (specifically associated with the causal propagation of disturbances). That is, one has a supervenience relation between any two families of properties, one for the particle and one for the field. In general, one set of properties, A, can be said to supervene on another set of properties, B, known as the base set, if and only if there is an appropriate relationship between each A-property and another property that can be constructed from the B-properties (cf. [28], passim), satisfying conditions laid out below.

Because the sets of properties involved in the relationship between particles of particle physics and fields of quantum field theory are those of the particles (A) and the quantum fields (B) discussed above, one finds supervenience of particles on fields by virtue of the supervenience of particle properties on field properties: In the current case, the base properties, B, are field properties, including mass and spin (or helicity for massless particles) and the set A of particle properties indicated by the Wigner classification including, in particular, mass and spin. Elementary particles are units like what Weinberg call “bundles”, as noted in the Introduction, also possessing energy-momentum. Crucially, as illustrated in the example of the previous section, because the energy-momentum is strictly conserved, the mass appearing in the field propagator is variable and so will differ, in general, from the fixed values of mass for the particle identified via the Wigner classification as shown in the previous section; this in itself precludes the reduction of particles to fields even though the energy-momentum values of the two entities agree.

The supervenience relation here is one characterized by three conditions: (i) covariance, (ii) dependence, and (iii) non-reducibility ([28], Ch. 1); in particular, it is the correspondence of the defining properties of both particles and the fields. *Covariance* is the requirement that variations in A properties are correlated with variations in B properties. The correspondence in the RQFT formalism of the particle to the field can be made via the propagator, which contains the common properties of mass and spin (or helicity): A correlation is found by the form of the propagator (cf. Equation (9)), in that the spin (or helicity) appearing in this field-theoretical function is exactly, i.e., perfectly correlated to that of the particle and, for the *other* defining property, mass, the greater contributions to the likelihood of the transitions between (incoming and outgoing) states resulting from interactions are due to those for which the mass value in the propagator (a B property) is nearer the rest mass (an A property), where the denominator of the propagator, which is proportional to the difference between the two, is smallest. If detected, the arrival time of the particle measured will be similarly correlated with the expected propagation time. *Dependence* can be seen directly in the appearance of the field’s B-properties in the propagator, cf. Equation (9), of the properties of the particle (A-properties), along with a corresponding energy-momentum (e.g., via the Feynman rules when calculating state transitions involved scattering events via perturbation theory) attributable to the particle. *Non-reducibililty* is seen in the previous section in the generally many-to-one relation of the field properties (appearing in the general propagator) to particle properties—in particular, in the case of *mass*, which ranges over a set of values (with units of mass) whereas the mass of the particle, $m_{\mathrm{rest}}$, is unique and fixed. With the basic quantum-field-theoretical elements related to particles physics laid in the preceding sections, the supervenience of particles on fields, rather than the reduction of the former to the latter, is evident. (Notably, regarding this, Anthony Duncan makes the following comment. “At this point a characteristic (and for beginning students, frequently baffling) feature of LQFTs [Local Relativistic Quantum Field Theories] becomes apparent: namely, the absence of any preferred, one-to-one connection between particles and fields.” [21], p. 61).

Elementary particles supervene on fields as discrete, compresent units (sets of properties, necessarily including mass, spin, and energy-momentum, and contingently various charges); although they have space-time propagation possibilities described by the field-disturbances integrated over all space-time and involving the same properties, this propagation is not over strictly spatially localized regions or as distinct plane waves that correspond to free-field quanta—as just noted, the values of all the above properties do not necessarily correspond in a one-to-one relation. Two particles are identical in species by virtue of their rest mass, spin, and charges, which are dependent on the field properties as indicated in the field propagators, while those propagators, with which they are correlated, involve a range of masses differing from the rest mass. Although the corresponding particle mass is not reducible to the propagator mass during interaction, the field possesses the same space-time symmetry and their common properties satisfy the same conservation laws as the particle with the propagation determined by its propagators and, in the zero-interaction interaction limit, the mass of the propagator approaches the corresponding particle rest mass $m_{\mathrm{rest}}$ as closely as required, providing a dependence of the rest mass on the mass appearing in the propagator, as expected given that it is in that limit that the particle is closest to being isolated.

The compresence of properties forming a particle unit is consistent with the field behavior, in that the conserved quantities are indicated by Noether’s theorem which implies the existence of conserved quantities arising from the conserved currents corresponding to symmetry under the continuous translations of the *field* in space-time: When the energy-momentum (tensor) is integrated over space one obtains the field total energy and total momentum with the existence of these conserved ‘currents’ implying that energy and momentum flow locally, and symmetry under Lorentz boost implies a local flow of conserved angular momentum even if it is not localized. Relevant internal symmetries similarly given rise to local flow of conserved (e.g., electric) charges; cf., e.g., [14], Ch. 3. The unified character of the flow of properties does not require any specific space-time location but only a co-appearance of defining properties upon measurement enforced by conservation laws, for example, for momentum as imposed in calculations via in the Feynman rules for identifying and properly accounting for scattering amplitudes. The dependence on the field, for example, of the force-mediate particles is exhibited through the field propagators in the standard perturbative treatment of quantum processes in which scattering probabilities are calculated. The transition probabilities for scattering are standardly calculated using the LSZ reduction formula

(14) $$\langle f|S|i\rangle \propto \langle \Omega |T\left\{\phi \left(x_{1}\right)\phi \left(x_{2}\right)\ldots \phi \left(x_{n}\right)\right\}|\Omega \rangle ,$$

where the $\phi (x_{i})$ are the operators corresponding to the interacting fields at space-time points $x_{i}$ (cf., e.g., [29], Section 6.1). This is most evident in the case of interactions of second-order in *g*, for example in QED, which involves a single intermediary particle and so a simple propagator such as that of Equation (7) which appears in the Feynman rules for calculating the right-hand side of Equation (10).

## 6. Conclusions

The traditional notion of the elementary particle as a simple free-field excitation of a quantum field fails to be valid in any physical situation in which interactions are taking place; due to the omnipresence of interactions between particles, a harmonic-wave description of field propagation can only approximately apply to what are very nearly free fields. Under general circumstances, field behavior for its part is only well described generally by less orderly distributions, for example, the field propagators appearing in the probability amplitudes of the perturbation-theoretical method of predicting the particle-state transitions of scattering processes. However, the set of properties corresponding to allowed irreducible Poincaré group representations, which has thus far been considered only as a means of distinguishing particle *classes*, is sufficient to provide the means to indicate the properties of a particle, which is seen to propagate according to such a description and *supervene* on its corresponding field: Because the mass appearing in the field propagator underlying the particle can vary, particles are only dependent on, not reducible to, their associated fields and definable as a unified collection of its mass, spin, consistently possessing other associated, conserved properties as well. At the end of scattering interactions, when measured for, discrete such unified collections of properties are observed.

In general, such particles interact with one another via other particles and depend on underlying field disturbances which, however, go “off-mass-shell” during interaction as can be seen in the indeterminacy of energy within the finite times of individual interaction events. The flux of particles is, nonetheless, accounted for in accordance with conservation laws for mass-energy, spin, and the charges involved. One sees that both fields *and* particles exist and jointly constitute the ontology of relativistic interacting field theories such as those of the Standard Model of particle physics. As Schwinger commented of elementary particles as described by QFT, “[I]n the real world, the localized excitation represented by an electromagnetic field, for example, does not just create a photon; it transfers energy, momentum and angular momentum, and then Nature goes to work. And so, it may create a photon, or an electron–positron pair, or anything else with the right quantum numbers. The various Green’s functions are the correlation functions among such localized excitations, and the study of their space-time behavior is the instrument for the identification of the physical particles and of their interactions” ([30], pp. 347–348). It is just this “work” that is captured through the mathematical tools considered here and, when describable via scattering theory, is explicable in terms of particles whose behavior is dependent on that of the fields on which they supervene.
